# Impact of the partnership between Japanese Society of Gastroenterological Surgery, Society of Surgical Oncology, and *Annals of Surgical Oncology*

**DOI:** 10.1002/ags3.12259

**Published:** 2019-05-18

**Authors:** Kelly M. McMasters

**Affiliations:** ^1^ The Hiram C. Polk Jr., MD Department of Surgery University of Louisville School of Medicine Louisville USA

**Keywords:** educational lecture, Japanese Society of Gastroenterological Surgery, Society of Surgical Oncology

## Abstract

The Society of Surgical Oncology (SSO) and its official journal, *Annals of Surgical Oncology*, have had a very important and successful partnership with the Japanese Society of Gastroenterological Surgery (JSGS). This partnership has advanced our missions of promoting high‐quality surgical science focused on improving patient outcomes.

The Society of Surgical Oncology (SSO) and its official journal, *Annals of Surgical Oncology*, have had a very important and successful partnership with the Japanese Society of Gastroenterological Surgery (JSGS). As recent President of the SSO and now Editor‐in‐Chief of *Annals of Surgical Oncology*, I would like to express our appreciation and gratitude for this longstanding relationship. This partnership has advanced our missions of promoting high‐quality surgical science focused on improving patient outcomes.

*Annals of Surgical Oncology* is a global journal. It has a worldwide readership and an international Editorial Board. It receives articles from all over the world. More than 10 000 institutions around the globe have access to *Annals of Surgical Oncology*. We expect that we will exceed 1 million article downloads this year, which reaches an enormous number of readers around the globe.

We celebrated the 25th anniversary of *Annals of Surgical Oncology* at the 2018 SSO Annual Meeting. Success of the Journal can largely be credited to the efforts of Dr Charles Balch. Dr Balch was the founding Editor‐in‐Chief and served for 25 years. He has often been a visiting professor to Japan, and has attended the JSGS meeting many times. The most prestigious award bestowed by the SSO is the Distinguished Service Award; Dr Balch is a former recipient. In 2018, the SSO renamed this award the Charles Balch Distinguished Service Award in recognition of Dr Balch's many contributions to make the SSO a global society and *Annals of Surgical Oncology* a global journal.

Article downloads from *Annals of Surgical Oncology* have steadily increased from 161 000 in 2007 to nearly 1 million article downloads in 2017 (Figure 1). The number of downloads and worldwide access to the Journal truly reflects its international prominence. Although 34% of the downloads were from North America, 32% were from the Asia‐Pacific region, 24% were from Europe, and 10% were from the rest of the world (Figure 2). It is important to note that of all the surgical journals in the world, *Annals of Surgical Oncology* has more published articles, more published pages, and more downloads than any other surgery journal.

**Figure 1:**
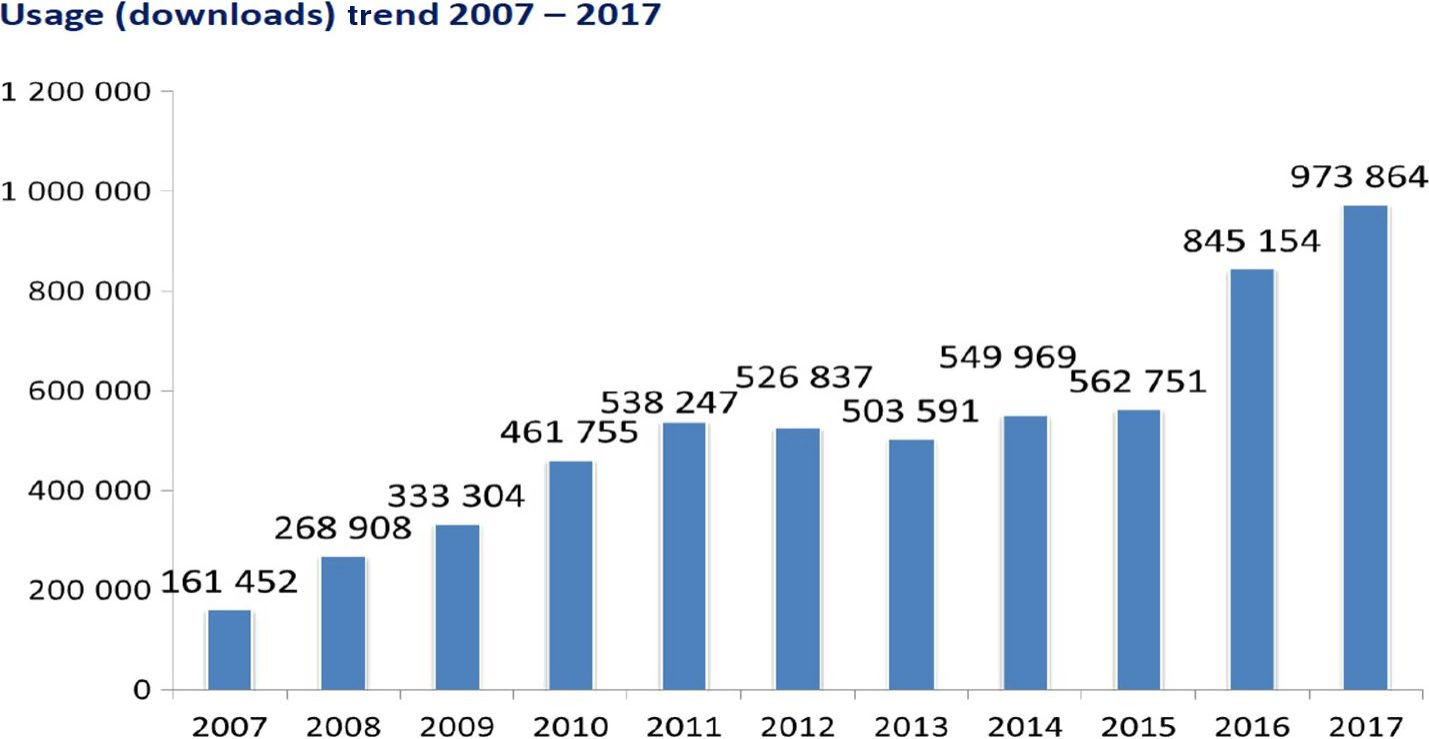
*Annals of Surgical Oncology* downloads over time

**Figure 2:**
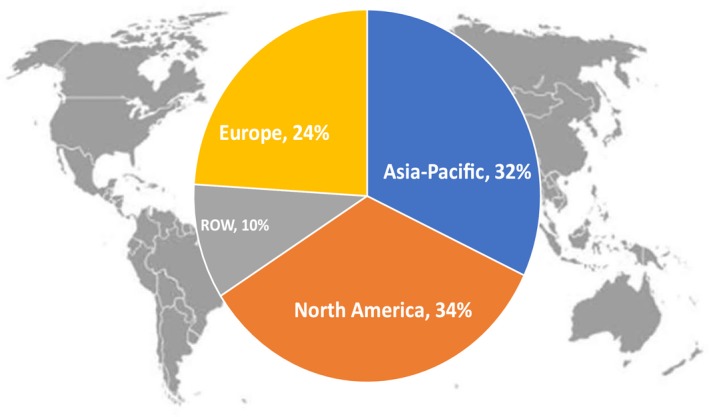
*Annals of Surgical Oncology* article submissions from around the world. ROW, rest of world

*Annals of Surgical Oncology* has global engagement in all areas, including readership, article submissions, and Editorial Board membership. We have had well over 2000 reviewers in the past decade who have participated in improving the scientific quality of the journal—many from Japan. Japanese members serve on the Editorial Board for several sections of *Annals of Surgical Oncology*, including breast oncology, colorectal cancer, gastrointestinal cancer, hepatobiliary tumors, thoracic oncology, and translational research and biomarkers.

Many of our best articles come from Japan. There has been a strong track record of submissions from authors in Japan—especially from the JSGS (Figure 3). In past years, *Annals of Surgical Oncology* published a supplement specifically devoted to the best articles from the JSGS meeting. The SSO and *Annals of Surgical Oncology* are very proud of this relationship.

**Figure 3:**
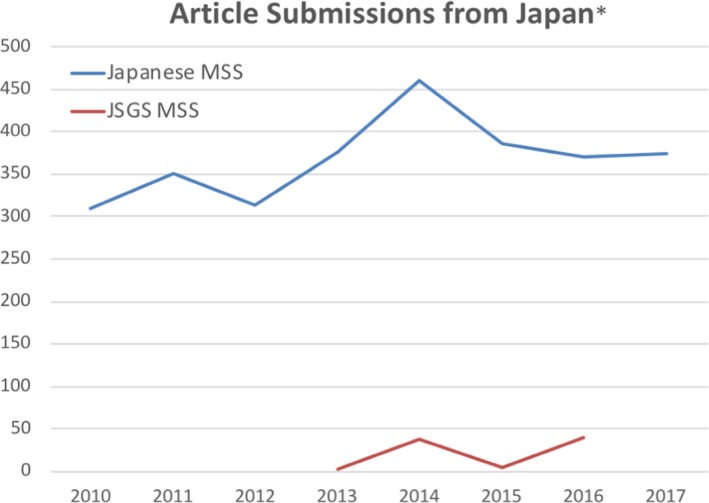
Strong record of *Annals of Surgical Oncology* manuscript submissions from Japan. MSS, manuscript submissions. *Original and revised submissions

Japanese surgeons, and JSGS members in particular, have a very strong record of manuscript acceptances. In 2017, 18% of the articles accepted in *Annals of Surgical Oncology* came from Japan and 39% came from the USA (Figure 4). This number is growing and we anticipate it will continue to improve as our partnership continues.

**Figure 4:**
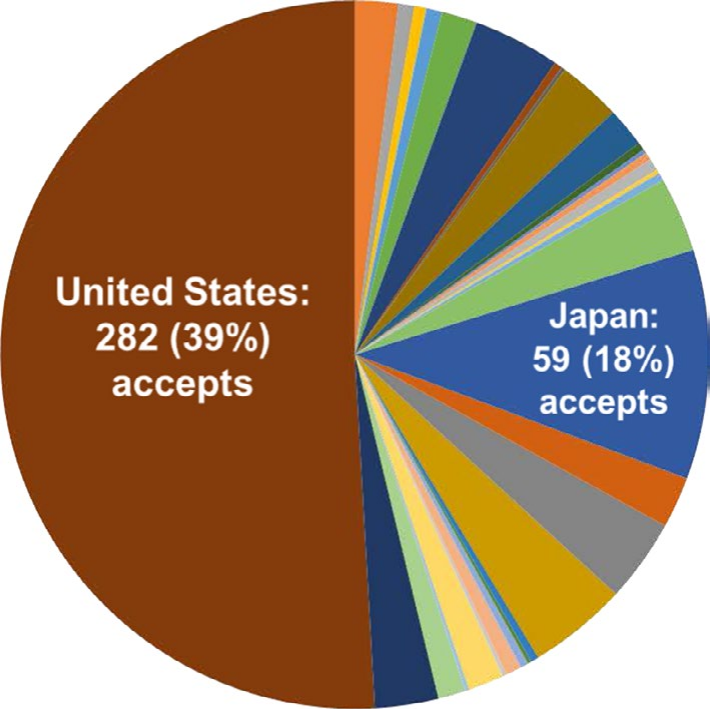
Japan has the second highest rate of manuscript acceptances to *Annals of Surgical Oncology* of all countries in the world

Of the Japanese articles published in *Annals of Surgical Oncology*, 81% are cited. In fact, they are well cited. The impact factor of articles published by Japanese authors has somewhat exceeded the average from the rest of the world. Another indicator of impact for groups of articles is the Category Normalized Citation Impact (CNCI). This rating is normalized for subject focus, age, and document type. A CNCI value of 1 represents performance on par with the world average, values above 1 are considered above average and values below 1 are considered below average. Figure 5 shows that articles authored by Japanese experts and published in *Annals of Surgical Oncology* have a higher CNCI than those Japanese authors’ articles published in all other surgical journals. When you publish in *Annals of Surgical Oncology*, more people have access to your articles, and more people will download, read, and cite your articles.

**Figure 5:**
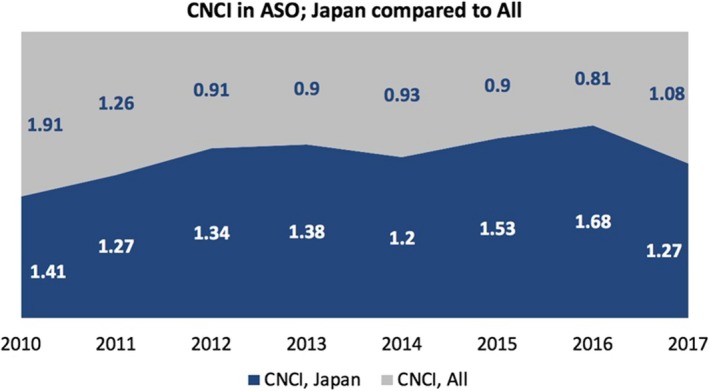
Annals of Surgical Oncology (ASO); Category Normalized Citation Impact (CNCI)

The most cited articles from JSGS were by Beppu et al^1^, Yamada et al^2^, Arita et al^3^, Terashima et al^4^, Kobayashi et al^5^, and Koyama et al.^6^ In fact, the most cited article for the entire journal—not just Japanese publications but for all of *Annals of Surgical Oncology* for 2016—was an article that we celebrated at the 2018 Annual Meeting of the SSO entitled: “Prognostic Significance of Neutrophil‐to‐Lymphocyte Ratio and Platelet‐to‐Lymphocyte Ratio in Oncologic Outcomes of Esophageal Cancer: A Systematic Review and Meta‐analysis” by Yodying et al.^7^ There were 41 citations and 1300 downloads of this article.

The most prolific author in *Annals of Surgical Oncology* is Professor Masaki Mori, who has also served on the Executive Council of SSO (Figure 6). He has 76 publications which have been cited 1128 times. We thank Professor Mori for his many contributions to the Journal and to the SSO.

**Figure 6:**
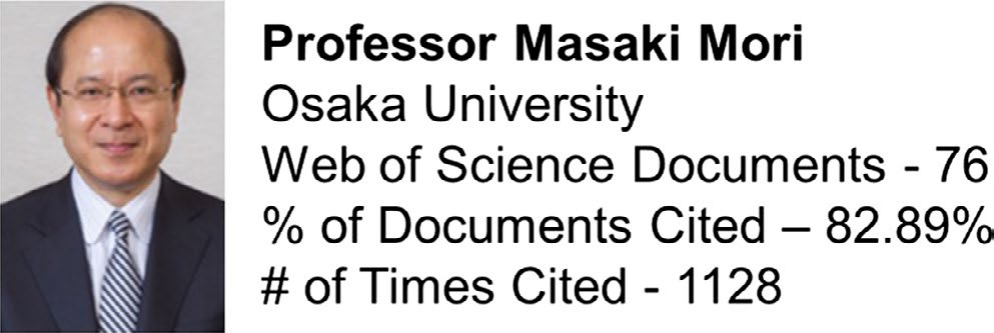
Professor Masaki Mori, the most prolific author in *Annals of Surgical Oncology*

The SSO strives to be the premier organization for surgeons dedicated to the multidisciplinary care of cancer patients and advancing the science, education and practice of cancer surgery. We understand that, as cancer surgeons, despite all of the advances in chemotherapy, targeted therapy, immunotherapy, and radiation therapy, often the single best thing that we can do for our cancer patients is to carry out a good cancer operation. We have learned to carry out cancer surgery more safely, less invasively, and with less morbidity. Yet, we know that surgery alone will not cure many of our patients because their disease has already progressed beyond what we can resect. That is why it is so important that we engage not only in research about surgical techniques, but we must also be involved in basic and translational research and development of adjuvant therapies, especially new targeted therapies and immunotherapies. This is what will allow us to improve patient outcomes and cure more patients in the future. The JSGS has been an important partner in advancing this research mission.

We believe that the SSO truly is a global society and we are very interested in increasing our global outreach. The SSO now has over 575 international members across all categories. There are many active members from Japan and this number is growing, as we attract more and more members from around the world to join the SSO and attend the Annual Meeting.

The SSO now has a new learning management system, ExpertEd@SSO, an online system that is going to continue to allow delivery of educational content to members around the world. Even if members are unable to attend the annual SSO meeting, this is a benefit of membership that is very important, as many people now learn on their phones and computers. A host of new educational offerings is constantly under development.

Recently, Dr Chandra Are established the Global Forum of Cancer Surgeons at the annual SSO meeting. The SSO plans to continue this as a major initiative to address global problems in cancer surgery, including educational concerns. Professors Mori and Eguchi were co‐authors who have contributed to this effort.^8^

At the last SSO meeting, 20 different countries were represented on the podium. SSO also offers global partner poster sessions, where partner societies are invited to present their top abstracts from their respective surgical oncology meetings at the SSO Annual Cancer Symposium. JSGS participated in 2017 and 2018. The SSO/JSGS Joint Symposium in 2017 on intraductal papillary mucinous neoplasms of the pancreas was also very successful.

The SSO and JSGS have also partnered in many career development activities, including the international career development exchange, which began in 2014. Several attendees from Japan have participated and this has been a very successful program. SSO members have also participated in observerships offered by JSGS. SSO members greatly value this opportunity.

In summary, *Annals of Surgical Oncology* is the most cited surgical journal in the world. We certainly want to encourage manuscript submissions from all of our global partners and especially from the JSGS. The Journal now offers simultaneous publication at the time of the SSO Annual Meeting for all of the top‐ranked papers. Papers from our partners at JSGS are always among the top‐tier submissions, so we continue to encourage your participation in the SSO program.

SSO will continue to lead the global dialogue for surgical oncology and promote the value of cancer surgeons. Many educational resources are available to SSO members. In addition to access to *Annals of Surgical Oncology*, there are many other educational offerings, including the SSO virtual meeting, webinars, and other materials available at ExpertEd@SSO. Many articles published in *Annals of Surgical Oncology* are published as open access articles and are freely available.

On behalf of the SSO and *Annals of Surgical Oncology*, thank you for the tremendous friendship and partnership between our organizations. We hope that this will only continue to grow stronger in the years ahead.

## DISCLOSURE

Conflicts of Interest: The author is the Editor‐in‐Chief of *Annals of Surgical Oncology*.

## References

[1] Beppu T , Miyamoto Y , Sakamoto Y , Imai K , Nitta H , Hayashi H , et al. Chemotherapy and targeted therapy for patients with initially unresectable colorectal liver metastases, focusing on conversion hepatectomy and long‐term survival. Ann Surg Oncol. 2014;21(Suppl 3):S405–S413. 24570379 10.1245/s10434-014-3577-x

[2] Yamada S , Utsunomiya T , Morine Y , Imura S , Ikemoto T , Arakawa Y , et al. Expressions of hypoxia‐inducible factor‐1 and epithelial cell adhesion molecule are linked with aggressive local recurrence of hepatocellular carcinoma after radiofrequency ablation therapy. Ann Surg Oncol. 2014;21(Suppl 3):S436–S442. 24566861 10.1245/s10434-014-3575-z

[3] Arita J , Ono Y , Takahashi M , Inoue Y , Takahashi Y , Saiura A . Usefulness of contrast‐enhanced intraoperative ultrasound in identifying disappearing liver metastases from colorectal carcinoma after chemotherapy. Ann Surg Oncol. 2014;21(Suppl 3):S390–S397. 24570378 10.1245/s10434-014-3576-y

[4] Terashima M , Tanabe K , Yoshida M , Kawahira H , Inada T , Okabe H , et al. Postgastrectomy Syndrome Assessment Scale (PGSAS)‐45 and changes in body weight are useful tools for evaluation of reconstruction methods following distal gastrectomy. Ann Surg Oncol. 2014;21(Suppl 3):S370–S378. 24590434 10.1245/s10434-014-3583-z

[5] Kobayashi H , West NP , Takahashi K , Perrakis A , Weber K , Hohenberger W , et al. Quality of surgery for stage III colon cancer: comparison between England, Germany, and Japan. Ann Surg Oncol. 2014;21(Suppl 3):S398–S404. 24566862 10.1245/s10434-014-3578-9

[6] Koyama M , Murata A , Sakamoto Y , Morohashi H , Takahashi S , Yoshida E , et al. Long‐term clinical and functional results of intersphincteric resection for lower rectal cancer. Ann Surg Oncol. 2014;21(Suppl 3):S422–S428. 24562938 10.1245/s10434-014-3573-1

[7] Yodying H , Matsuda A , Miyashita M , Matsumoto S , Sakurazawa N , Yamada M , et al. Prognostic significance of neutrophil‐to‐lymphocyte ratio and platelet‐to‐lymphocyte ratio in oncologic outcomes of esophageal cancer: a systematic review and meta‐analysis. Ann Surg Oncol. 2016;23(2):646–654. 26416715 10.1245/s10434-015-4869-5

[8] Are C , Coit DG , McMasters KM , Giuliano AE , Anderson BO , Balch CM , et al. Global forum of cancer surgeons: declaration of intent. Ann Surg Oncol. 2017;24(9):2429–2431. 28676922 10.1245/s10434-017-5951-y

